# Promoter-like sequences regulating transcriptional activity in neurexin and neuroligin genes

**DOI:** 10.1111/jnc.12372

**Published:** 2013-08-21

**Authors:** Fabian Runkel, Astrid Rohlmann, Carsten Reissner, Stefan-Martin Brand, Markus Missler

**Affiliations:** * Institute of Anatomy and Molecular Neurobiology, Westfälische Wilhelms-UniversityMünster, Germany; † Institute of Sports Medicine Molecular Genetics of Cardiavascular Disease, University Hospital MünsterMünster, Germany

**Keywords:** CpG island, MeCP2, neurexin, neuroligin, promoter, transcriptional activity

## Abstract

Synapse function requires the cell-adhesion molecules neurexins (Nrxn) and neuroligins (Nlgn). Although these molecules are essential for neurotransmission and prefer distinct isoform combinations for interaction, little is known about their transcriptional regulation. Here, we started to explore this important aspect because expression of *Nrxn1-3* and *Nlgn1-3* genes is altered in mice lacking the transcriptional regulator methyl-CpG-binding protein2 (MeCP2). Since MeCP2 can bind to methylated CpG-dinucleotides and *Nrxn*/*Nlgn* contain CpG-islands, we tested genomic sequences for transcriptional activity in reporter gene assays. We found that their influence on transcription are differentially activating or inhibiting. As we observed an activity difference between heterologous and neuronal cell lines for distinct *Nrxn1* and *Nlgn2* sequences, we dissected their putative promoter regions. In both genes, we identify regions in exon1 that can induce transcription, in addition to the alternative transcriptional start points in exon2. While the 5′-regions of *Nrxn1* and *Nlgn2* contain two CpG-rich elements that show distinct methylation frequency and binding to MeCP2, other regions may act independently of this transcriptional regulator. These data provide first insights into regulatory sequences of *Nrxn* and *Nlgn* genes that may represent an important aspect of their function at synapses in health and disease.

Neurons adapt their molecular equipment during development and activity-induced plasticity processes through transcriptional regulation (Cohen and Greenberg 2008), including the mostly presynaptic cell-adhesion molecules neurexins (Rozic-Kotliroff and Zisapel 2007; Iijima *et al*. 2011; Rozic *et al*. 2013). All three neurexin genes (*Nrxn1-3*) are presumably transcribed from two independent promoters that lead to the generation of extracellularly longer α-Nrxn and shorter β-Nrxn variants (Missler *et al*. 2011). In addition to these six major variants, extensive usage of five alternative splice sites in α-Nrxn, and two in β-Nrxn, may lead to more than 3000 isoforms (Rowen *et al*. 2002; Tabuchi and Südhof 2002), which were shown to be differentially distributed at the mRNA level (Ullrich *et al*. 1995). Nrxn play important roles in synaptic transmission and maturation of contacts (Missler *et al*. 2003; Graf *et al*. 2004), and some functions require *trans*-synaptic complex formation with neuroligin (Nlgn) (Ichtchenko *et al*. 1995; Boucard *et al*. 2005).

Studies in Nrxn knockout (KO) mice demonstrated decreased Ca^2^ ^+^-dependent synaptic release at excitatory and inhibitory synapses (Missler *et al*. 2003; Kattenstroth *et al*. 2004), and Nlgn KO mice similarly revealed an impaired synaptic transmission (Varoqueaux *et al*. 2006; Poulopoulos *et al*. 2009). Interactions of Nrxn with Nlgn, but also with other extracellular binding partners as LRRTM2, cerebellin, or dystroglycan (Missler *et al*. 2011), depend strongly on the molecular variants involved, and a code has been proposed for their binding affinities (Boucard *et al*. 2005; Chih *et al*. 2006; Koehnke *et al*. 2010). In addition, some Nlgn variants are localized to subtypes of synapses, with Nlgn1 restricted to excitatory (Song *et al*. 1999), Nlgn2 and Nlgn4 to inhibitory synapses (Varoqueaux *et al*. 2004; Hoon *et al*. 2011), while Nlgn3 occurs at both types (Budreck and Scheiffele 2007). Such regulation of localization and usage can be functionally relevant because Nlgn2 variants, for example, associated with inhibitory synapses, preferentially bind to α-Nrxn and this molecule, in turn, induces GABAergic post-synaptic differentiation (Graf *et al*. 2004; Chih *et al*. 2006).

Despite the wealth of information on their physiological roles and their link to autism-spectrum disorders and schizophrenia (reviewed in Südhof 2008), very little is known about the transcriptional regulation of *Nrxn* and *Nlgn* genes. Here, we observe that *Nrxn*/*Nlgn* expression is altered in brains of mice lacking the methyl-CpG-binding protein 2 (MeCP2), a transcriptional regulator in brain (Chahrour *et al*. 2008) and the cause of human Rett syndrome (Amir *et al*. 1999). Our strategy to start the analysis of *Nrxn*/*Nlgn* gene regulation in MeCP2 KO mice was based on the following considerations: (i) MeCP2 regulates gene expression dependent on DNA methylation (Shahbazian *et al*. 2002a; Kriaucionis and Bird 2003; Chang *et al*. 2006; Yasui *et al*. 2007), and *Nrxn*/*Nlgn* genes contain CpG-rich sequences; (ii) Rett syndrome is an autism-spectrum disorders (Chahrour and Zoghbi 2007), and MeCP2 KO mice that recapitulate symptoms of patients (Guy *et al*. 2001; Shahbazian *et al*. 2002a) suffer from impaired synaptic transmission (Chao *et al*. 2007, 2010; Medrihan *et al*. 2008), similar to the Nrxn/Nlgn mouse models. In this study, we identify regulatory sequences in *Nrxn* and *Nlgn* genes, some but not all containing CpG-islands, which are able to change reporter gene expression in heterologous and neuronal cell lines. In addition, we characterize novel transcription start sites and genomic fragments in *Nrxn1* and *Nlgn2* that depend on a distinct methylation frequency for their transcriptional activity. Thus, our study provides the first experimental data on the regulation of *Nrxn* and *Nlgn* expression at the transcriptional level.

## Materials and methods

### Animals

MeCP2 KO mouse strain *B6.129P2(C)-Mecp2 tm1.1Bird* was obtained from Jackson Laboratory (Bar Harbor, ME, USA), and maintained on a C57Bl/6J background as described (Medrihan *et al*. 2008). The animals were housed and bred according to the institutional and German governmental guidelines for animal welfare. All experiments were performed on hemizygous males (KO = MeCP2^−/y^) and their age-matched littermate controls (WT = MeCP2^+/y^). Mice were deeply anesthetized using isoflurane, decapitated, and brains removed to −80°C until further use. The experimental procedures strictly followed governmental regulations of animal welfare and were approved by the Institutional Animal Care and Use Committee (ZTE) of the Medical Faculty of the Westfälische Wilhelms-University, Münster, Germany. Mice were housed in a 12 h light–dark cycle in stable conditions of temperature and with access to food and water *ad libitum*.

### Real time qPCR analyses

Total brain RNA was isolated from day P7 and day P20 old MeCP2^−/y^ and wild-type mice with RNAzol-reagent (WAK-Chemie Medical, Steinbach, Germany). RT-qPCR using the LightCycler® 480 SYBR Green I Master protocol (Roche diagnostics, Mannheim, Germany) was performed with gene-specific primers (Table S1). For internal control, beta-actin reactions were used as a standard. All reactions for each gene (for WT and KO of P7 and P20) and corresponding control reactions were performed on a single 96-well plate. Quantitative standard reactions showed only minimal variation in qPCR-conditions between the plates. Each CT-value of a gene-specific reaction was calculated relative to the beta-actin control. Expression levels in WT samples were set to 1, and expression in MeCP2 KO calculated as x-fold expression of WT.

### Quantitative immunoblots

Immunoblots were performed with Triton-X100 lysates of brains from MeCP2 KO and WT mice at P7 and P20. Lysates of three animals per genotype were pooled and analyzed in duplicates on 12% sodium dodecyl sulfate–polyacrylamide gel electrophoresis gels. After blotting, membranes were incubated with antibodies Nlgn1-975, Nlgn2-799 or Nlgn3-804 against Nlgn isoforms (Synaptic Systems, Göttingen, Germany) at a concentration of 1 : 500 to 1 : 1000 in blocking solution at 4°C overnight. After incubation with horseradish peroxidase-conjugated secondary goat-anti-rabbit antibody (1 : 10 000; Biorad, Munich, Germany), the blots were developed with Immobilon chemiluminescense solution (Millipore, Schwalbach, Germany). Luminescense was measured and gray levels for each sample calculated. For loading control, all blots were re-incubated with an antibody against HSP70 (1 : 5000; Abcam, Cambridge, UK). Protein levels were normalized to HSP70 expression, and each Nlgn was probed on at least four independent blots.

### Cell cultures

PC12 cells (DSMZ, Braunschweig, Germany) were cultured in suspension using RPMI1640 media (Life Technologies, Darmstadt, Germany) supplemented with 10% horse-serum and 5% fetal calf serum (FCS). Transient transfection of PC12 cells was performed with 800 000 cells/6 wells in suspension, using Lipofectamine™2000 (Invitrogen, Karlsruhe, Germany) according to the suppliers protocol. To induce differentiation, PC12-cells were seeded at 150 000 cells/6-well coated with rat collagen (Roche Diagnostics), and treated the next day by addition of 50 μg/ml nerve growth factor (NGF) (Harbor Bio-Products, Norwood, MA, USA) in medium. For 1 week, cells were fed with fresh NGF-containing media every second day. Transfection was performed 8 days after seeding using the Lipofectamine™2000 protocol described above. HEK293 cells (ECACC, Salisbury, England) were cultured in 10 cm dishes in Dulbecco's modified Eagle's medium (Sigma-Aldrich, Munich, Germany) supplemented with 10% FCS. Cells were seeded in six-well plates at a density of 150 000 cells/well and transiently transfected with calcium phosphate 1 day after plating.

### Luciferase reporter gene assays

Genomic sequences were amplified by PCR and cloned in pGL4.23(luc2/minP) vector (Promega, Mannheim, Germany). This vector contains a medium intrinsic promoter activity to drive the *firefly* luciferase, allowing us to study activating as well as inhibiting effects (Chahrour *et al*. 2008; Suter *et al*. 2011). The intrinsic promoter is located between cloning site and luciferase to impair direct effects from the inserted sequences. As control for transfection efficiency, cells were co-transfected with the pGL4.74 (hRluc/TK) vector (Promega) that expresses *renilla* luciferase constitutively, and empty pGL4.23 (luc2/minP) vector was used as an internal standard. Transfected cells were split in three (HEK293) or two (PC12) wells of a 96-well LUMITRAC 200-plate (Greiner Bio-one, Frickenhausen, Germany) after 48 h. Both luciferases were measured sequentially with the Dual-Glo™ system (Promega) on a CENTRO LB 960 luminometer (Berthold Technologies, Bad Wildbad, Germany) for 5 s at 20°C. After measurement of the luciferase activity, Stop-and-Glo™ solution was added to quench the *firefly* luciferase activity and to offer a substrate for the *renilla* luciferase. Luciferase activity for each well was divided by the renilla activity, a mean value calculated, and results were normalized to the activity of the pGL4.23 control vector. Activation of luciferase is expressed as values > 1, inhibition as values < 1.

To evaluate methylation in the *Nlgn2* gene, we performed additional luciferase assays with *in vitro*-methylated constructs containing the first and the second exon. Same amounts of DNA and conditions for the transfection of HEK, PC12, and differentiated PC12 cells were used as above but the test constructs and the empty control vector were methylated before with CpG Methylase (*M.SssI*) (Zymo Research, Freiburg, Germany).

### 5′-race

To identify the transcription start point (TSS) for the Nrxn1, we performed a 5′-RACE procedure (rapid amplification of cDNA ends) with FirstChoice® RLM-RACE (Invitrogen), using the following oligonucleotide primers: outer-primer, 5′-CTCAAACCCTGCTGTGAAGTAGC-3; inner-primer, 5′-GCCTGGTTATTCCCCTTAGC-3′; control primer: 5′-GAGACAGCTCCATTTTTCAATGC-3′. Resulting PCR fragments were sequenced and compared to the mouse genome assembly NCBI37/mm9 and to known transcripts with the BLAT genome search program at UCSC (http://genome.ucsc.edu/index.html). The resulting new 5′-mRNA sequences for Nrxn1α were submitted to GenBank (accession number KC747116, published June, 18th, 2013).

### Methylation-specific PCR

To detect methylated CpG-dinucleotides, we performed methylation-specific PCRs. Bisulfite conversion of 1 μg genomic DNA was performed with EZ DNA Methylation-Gold™ Kit (Zymo Research), and was derived from brain and liver tissue (for *Nrxn1*, Fig. 5), or from normal and differentiated PC12 cells (for *Nlgn2*, Fig. 8). Primers specific for methylated versus unmethylated sequences were selected with application MethPrimer (http://www.urogene.org/methprimer/index1.html), and PCRs performed with GoTaq®-Polymerase (Promega), using the following oligonucleotide primer pairs: (i) MSP-A methylated sense (5′-GGTATTGCGGAGAGTTTAGTTTC-3′) versus antisense (5′-TACTACTACCTTTTTCCGAAACGTC-3′); (ii) MSP-A unmethylated sense (5′- GGTATTGTGGAGAGTTTAGTTTTGT-3′) versus antisense (5′- TACTACTACCTTTTTCCAAAACATC-3′); (iii) MSP-B methylated sense (5′-TTAAGTTTAAGCGTAGGGATATGAC-3′) versus antisense (5′-CTATAACGAATTAACCCGAACGT-3′); (iv) MSP-B unmethylated sense (5′-AAGTTTAAGTGTAGGGATATGATGG-3′) versus antisense (5′- CCTATAACAAATTAACCCAAACATC-3′); (v) MSP1 methylated sense (5′-GCGGAAAGTAGTTTTTATTTCG-3′) versus antisense (5′-TCAACAAAAATTTTACAAAAACGTC-3′); (vi) MSP1 unmethylated sense (5′-GGTGGAAAGTAGTTTTTATTTTGG-3′) versus antisense (5′-TCAACAAAAATTTTACAAAAACATC-3′); (vii) MSP2 methylated sense (5′-TTTTTTTTATATTTTAGAGGGCGG-3′) versus antisense (5′-AATACTCAATAACCAAAAAACCCG-3′); (viii) MSP2 unmethylated sense (5′-TTTTTTTTATATTTTAGAGGGTGG-3′) versus antisense (5′-ATACTCAATAACCAAAAAACCCAAA-3′); (ix) MSP3 methylated sense (5′-AGTTTAATAACGAGATTTTGGGTTC-3′) versus antisense (5′-TAATTCTACACGTAAATAACGACCG-3′); (x) MSP3 unmethylated sense (5′-GTTTAATAATGAGATTTTGGGTTTG-3′) versus antisense (5′-ATTCTACACATAAATAACAACCACC-3′). PCRs were repeated 3–11 times on independent DNA samples and positive PCR reactions for methylated versus unmethylated primer pairs were counted.

### Chromatin-immunoprecipitation (ChIP) PCR

To test the interaction of MeCP2 with methylated genomic sequences in *Nrxn1* and *Nlgn2*, we performed ChIP-PCR using an anti-MeCP2 antibody (H-300; Santa-Cruz Biotechnology, Heidelberg, Germany). Genomic DNA was cross-linked and digested with the PIERCE® Chromatin Prep Module (Thermo Scientific, Karlsruhe, Germany) from half mouse brain or 6 × 10^6^ native or differentiated PC12 cells. IP was performed overnight in phosphate-buffered saline at 4°C and antibody-bound DNA recovered with protein-A sepharose (GE-Healthcare, Uppsala, Sweden). Beads were released in glycine buffer (pH 1.8), and DNA-bound proteins digested with Proteinase K in Tris-HCl (pH 9.0). DNA was purified by phenol/chloroform extraction and ethanol precipitation. PCRs were performed with GoTaq®-Polymerase (Promega) and the following primers pairs: (i) MSP-A-PCR sense (5′-AGTCTCCGCCCTAAGATTCC-3′) versus antisense (5′-TGAGGAAGGGAAGAACATGG-3′); (ii) MSP-B-PCR sense (5′- CTTGGCTGCAGTCCTTGC-3′) versus antisense (5′- GGACATGACGGTGTTCAGC-3′); (iii) MSP1-PCR sense (5′-CGGCGGATCATCACTCTCG-3′) versus antisense (5′- GCGGACGGGTTTCAGAAGG-3′); (iv) MSP2-PCR sense (5′-CCTCCAAGTTGTCGGTGAAC-3′) versus antisense (5′-CAATCAGCATGTGGCTCCT-3′). Additional control reactions were performed on untreated genomic DNA.

### Statistics

Each RT-qPCR, immunoblotting, luciferase activity, methylation-specific-PCR (MSP), and ChIP experiment was performed at least three times on independent samples, with exact *n* values given in the figure legends or supplemental tables. Data obtained were subjected to statistical analysis by Student's *t*-test using Prism 6 (Graphpad Software Inc., La Jolla, CA, USA); *p* value < 0.05 is considered significant, and significance levels denoted as outlined in the figure legends.

## Results

### *Nrxn* and *Nlgn* expression is altered in brains of MeCP2-deficient mice

**Figure 1:**
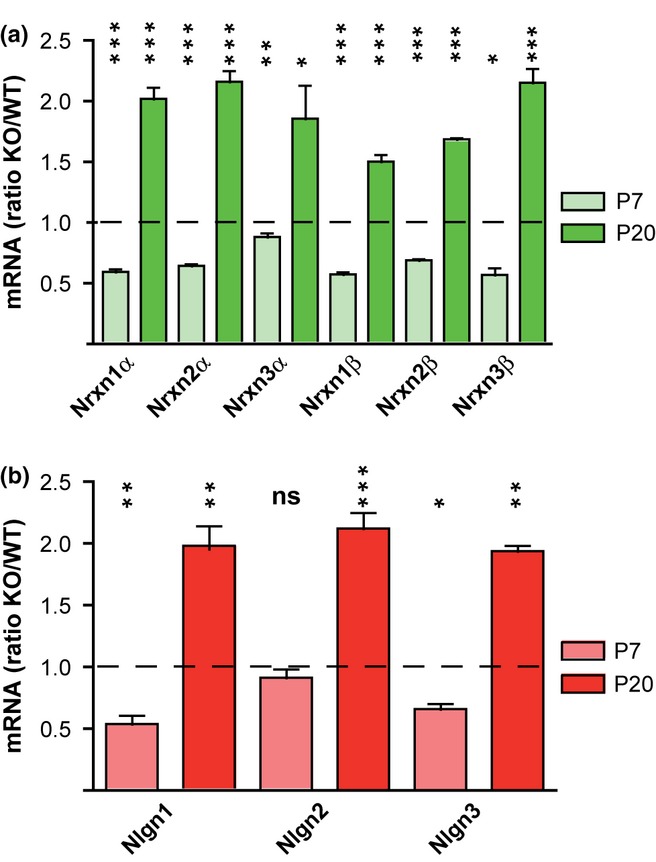
Expression of *Nrxn* and *Nlgn* genes in methyl-CpG-binding protein2 (MeCP2) knockout (KO) brains. (a) RT-qPCR analysis of α-Nrxn and β-Nrxn variants from *Nrxn1-3* in WT and MeCP2 KO brains at post-natal days (P) 7 (light green) and P20 (dark green). mRNA levels are expressed as a ratio of KO relative to WT (= 1, dashed line), revealing a decrease at day P7 and an increase at day P20 in KO. (b) Similar RT-qPCR analysis as in (a) for *Nlgn1-3* genes showing a similar tendency as for Nrxn (P7, light red; P20, dark red). Data are means ± sem (*n *=* *3–6 brains/genotype and age). Significance was determined by Student's *t*-test, significance levels are indicated as *p <* *0.05, **p <* *0.01, ***p <* *0.001, ns = non-significant.

Almost no information is available on the transcriptional regulation of *Nrxn* and *Nlgn* genes that are predominantly expressed in brain. To address this question, we tested if their expression involves MeCP2, a transcriptional regulator specific to the nervous system that affects a large number of target genes (Shahbazian *et al*. 2002b; Chahrour *et al*. 2008). Since we found impaired neurotransmission in MeCP2-deficient KO mice early postnatally (Medrihan *et al*. 2008), we first performed RT-qPCR analysis of WT and KO brains at post-natal day P7 to compare *Nrxn* and *Nlgn* mRNA levels (Fig. 1). For *Nlgn*, we restricted this analysis to *Nlgn1-3* because *Nlgn4* shows very limited expression in brain (Hoon *et al*. 2011). RT-qPCR experiments with oligonucleotide primers located on different exons (Table S1) revealed a 20–50% lower abundance of all *Nrxn* (Fig. 1a) and most *Nlgn* (Fig. 1b) transcripts in KO brains at P7. These data suggest that MeCP2 may act as an activator for these genes during the early post-natal period, consistent with an essential role of α-Nrxn and Nlgn in early synapse function (Missler *et al*. 2003; Varoqueaux *et al*. 2006). In addition, the observation that *Nrxn* and *Nlgn* were synchronously regulated, with the exception of *Nlgn2* that remained unchanged at this age (Fig. 1b), emphasizes that they are functionally linked molecules (Südhof 2008).

**Figure 2:**
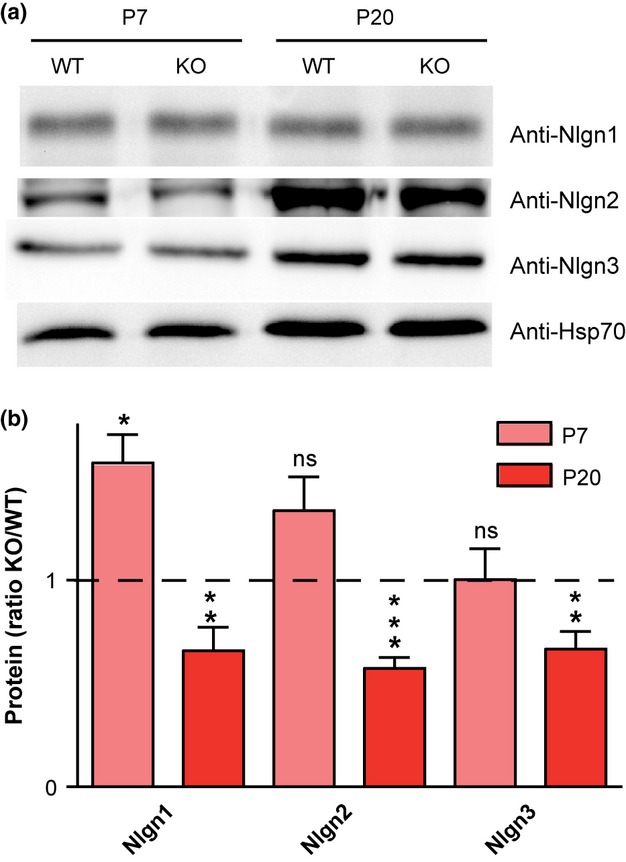
Nlgn protein levels in methyl-CpG-binding protein2 (MeCP2) knockout (KO) brains. (a) Immunoblots of brain lysates from WT and MeCP2 KO mice at P7 and P20 probed with antibodies specific to Nlgn1, -2 and -3, and HSP70 as control. (b) Quantification of protein levels of the three Nlgn variants in MeCP2 KO brains relative to WT samples (= 1, dashed line; 3–4 brains/genotype and age) at P7 (light red) and P20 (dark red). Bar graphs represent normalized protein levels from at least five independent experiments as shown in (a). Data are means ± SEM. Significance was determined by Student's *t*-test, significance levels are indicated as *p <* *0.05, **p <* *0.01, ***p <* *0.001, ns = non-significant.

To investigate if the expression levels of *Nrxn* and *Nlgn* change during post-natal development, we also investigated brain samples from P20. At this age, which marks the end of the rapid period of synapse formation in many areas, we found increased *Nrxn* (Fig. 1a) and *Nlgn* (Fig. 1b) transcript levels in MeCP2 KO brains. The relative abundance of all variants was elevated to 150–220% of WT (Table S1), suggesting that lower mRNA levels of *Nrxn*/*Nlgn* normally observed in adult brains (Irie *et al*. 1997) are because of active suppression by MeCP2. To test if the altered mRNA expression had an effect on protein levels, we performed quantitative immunoblot analysis for all three Nlgn in brain lysates of P7 and P20 mice (Nrxn could not be tested because of lack of isoform-specific antibodies). Although only the Nlgn1 signal was clearly elevated in KO early postnatally, the protein levels of all three Nlgn isoforms dropped significantly to about 60% in MeCP2 KOs at P20 (Fig. 2; Table S2). These data show proof-of-concept that changes in *Nrxn*/*Nlgn* mRNA expression can affect actual protein levels but at the same time caution against any simplistic interpretations because there was no linear correlation between mRNA and protein levels, consistent with previous studies on differentially expressed genes in MeCP2 KO mice (Shahbazian *et al*. 2002b). However, the reduction in Nlgn protein in older KO animals could reflect the overall decline of synapse numbers in MeCP2-deficient brains (Chao *et al*. 2007, 2010), possibly leading to a compensatory increase in mRNA expression (Fig. 1b).

### Regulatory sequences in *Nrxn* genes

Although it has become evident that MeCP2 can associate with DNA independent of CpG-islands (Yasui *et al*. 2007), its ability to bind to methylated CpG-dinucleotides is name giver for this transcriptional regulator (Skene *et al*. 2010), and prompted us to narrow the search for regulatory sequences in *Nrxn* and *Nlgn* genes. We predicted putative promoter regions and CpG-rich elements by *in silico* analysis, and then cloned and tested these genomic sequences using a dual luciferase reporter assay with intrinsic promoter activity that can be increased or decreased by inserted test sequences. In the reporter system chosen, the intrinsic promoter is located between luciferase and cloning site to avoid that exon/intron borders or UTR sequences directly influence the generation of the luciferase RNA.

**Figure 3:**
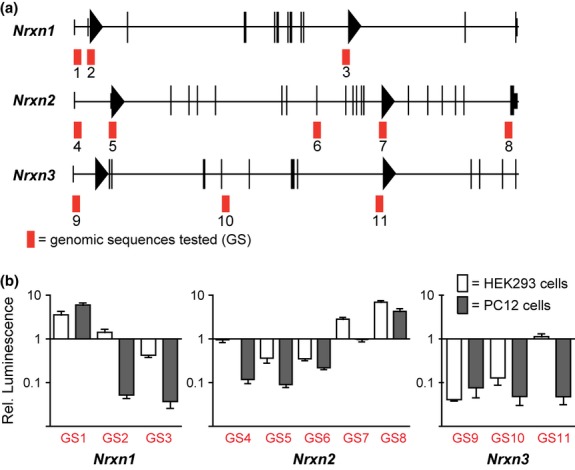
Regulatory sequences in *Nrxn1-3*. (a) Scheme showing exon/intron organization of all three murine *Nrxn* genes. Representation is to scale with the exception of size of exons (vertical black bars). Arrowheads indicate translation start of α-Nrxn (left) and β-Nrxn (right) variants. Regulatory sequences were predicted by *in silico* analysis and test regions cloned (red bars, GS1-11), including potential promoter regions upstream of transcriptional starting sites and/or CpG-rich sequences. (b) Results of a dual luciferase reporter gene assay in HEK293 (white bars) and PC12 cells (dark gray bars) used to determine regulatory activity of genomic test sequences (GS numbers as in a). Data are means ± SEM; values for bar graphs are in relation to control vector and blotted logarithmically (> 1 = activation, < 1 = inhibition).

Since *Nrxn* are among the largest genes in the mammalian genome (Tabuchi and Südhof 2002), we mostly focused on regions upstream of TSS: GS1 and GS2 for *Nrxn1α*, GS3 for *Nrxn1β*, GS5 for *Nrxn2α*, GS7 for *Nrxn2β*, GS9 for *Nrxn3α* and GS11 for *Nrxn3β* (Fig. 3a). Of these regions, GS2, GS3, GS5, and GS7 contain so-called CpG-islands. We also tested fragments elsewhere in the genes that were identified by their high CpG-content, including sequences from *Nrxn2* (GS4, GS6 and GS8) and *Nrxn3* (GS10) (Fig. 3a; detailed information on the genomic location, oligonucleotides and statistics in Table S3). Interestingly, most of the constructs displayed an inhibitory effect on the luciferase expression in both heterologous HEK293 cells and more neuron-like PC12 cells (Fig. 3b). The putative promoter regions of *Nrxn2* and *Nrxn3* (GS4 and GS9) down-regulated the reporter gene expression in both cell types, and the assumed *Nrxn1* promoter region (GS2) showed a particularly strong inhibition to 5% of the vector control in PC12 cells. A slightly more upstream located region of *Nrxn1* (GS1), however, produced an activating effect that was even stronger in PC12 cells (> 600%). Activation was also found in the 3′-regions of *Nrxn2* (Fig. 3b; GS7 and GS8), in which GS7 marks the transcription start point of the *Nrxn2β*-specific exon, but this construct was silent in PC12 cells. The construct GS8, consisting of sequences preceding the last exon of *Nrxn2*, displayed the strongest activity in both cell lines tested, in support of results that alternative splicing in the distal part of Nrxn influences activity-dependent regulation (Iijima *et al*. 2011).

**Figure 4:**
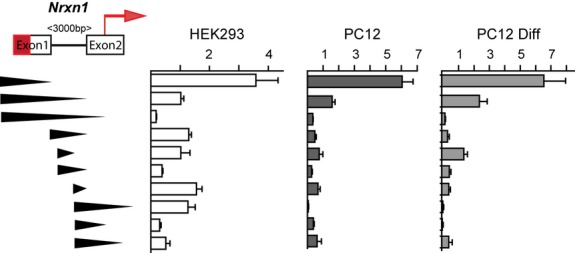
Dissection of transcriptionally active sequences in *Nrxn1*. Black arrowheads (left) represent position and length of genomic sequences cloned to identify the putative *Nrxn1α* promoter region. Fragments tested are shown in relation to exon1 and exon2 of the *Nrxn1* gene depicted above. Red arrow = translation start in exon2, red box in exon1 = novel transcription start site. Three groups of bar graphs show quantitative results of luciferase assays in HEK293 (white), undifferentiated PC12 (dark gray), and differentiated PC12 cells (light gray). Increased translational activity is restricted to sequences containing exon1. Data are means ± SEM (units: relative luminescence); values calculated in relation to control vector (= 1).

The differential effects of GS1 and GS2 from *Nrxn1* on expression are remarkable because the homology in this part is about 90% with a conserved exon/intron structure (Figure S1). Together with the differential distribution of CpG-islands between GS1 and GS2, we reasoned that the 5′-region of *Nrxn1* could present a model to study the role of MeCP2. Unfortunately, the most distal part of exon1 seemed to be missing in existing databases because only some brain-derived transcripts extend into exon1 (data not shown). To clarify this issue, we performed 5′-RACE experiments with the First choice™ RLM-RACE system. Using this approach, we identified a novel TSS in exon1 (Fig. 4, red box in exon1 of scheme), and then tested several overlapping genomic fragments that span these first two exons of *Nrxn1* in our luciferase assays (Fig. 4, arrowheads). In addition to HEK293 and undifferentiated PC12 cells, we also transfected PC12 cells that were differentiated by addition of NGF to evaluate the influence of neuronal maturation (Fig. 4; detailed information in Table S4). The results revealed that activation of expression is driven by sequences adjacent to exon1, and this activity is stronger in PC12 cells than in HEK293 and can further be enhanced by neuronal differentiation. Inclusion of sequences from the first intron and exon2 of *Nrxn1*, in turn, diminished the activating properties especially in the neuron-like cells (Fig. 4, light gray bars). Thus, our analysis demonstrates that *Nrxn* genes contain several regulatory sequences with and without CpG-islands, and raises the possibility that *Nrxn1* contains two neighbored regions differentially affected by MeCP2.

### Methylation of *Nrxn1* and MeCP2 association

**Figure 5:**
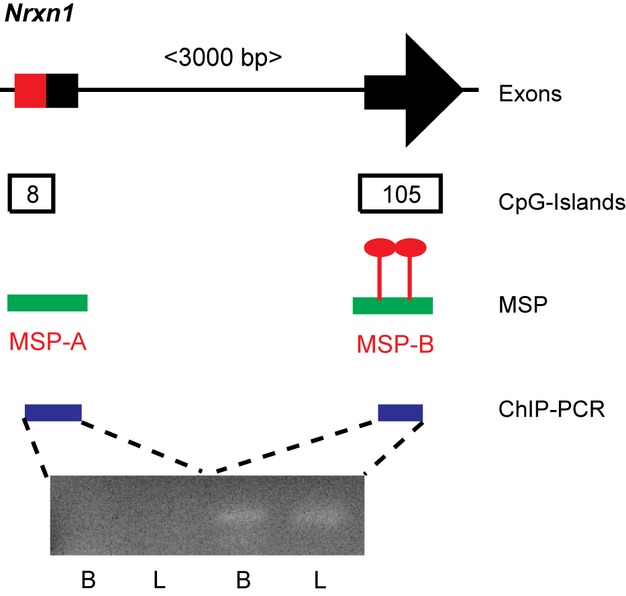
Methylation frequency and methyl-CpG-binding protein2 (MeCP2) binding at exon1 and exon2 sequences of *Nrxn1*. Schematic representation of the 5′-region of Nrxn1 (black arrowhead = translation start point, black boxes = UTR). Bioinformatics predict that exon1 and exon2 contain different numbers of CpG-dinucleotides (numbered boxes). Green lines represent PCR products used for methylation-specific PCRs (MSP) on bi-sulfite converted genomic DNA (red shapes indicate the relative methylation frequency observed). Blue lines denote position of PCR products amplified after chromatin-immunoprecipitation with anti-MeCP2 (ChIP-PCR), shown on sample gel images below (B = brain, L = liver tissue as templates). Positive signals of ChIP-PCR could only be found for MSP-B sequences (right lanes) that are more methylated than MSP-A sequences.

To explore the idea that expression can be differentially regulated from exon1 versus exon2 sequences of *Nrxn1* by MeCP2, we tested the methylation frequency of the CpG- elements in these exons (Fig. 5, boxed numbers) with MSP on genomic DNA from WT brain. Results of three independent MSP experiments show that exon1 sequences were not methylated (Fig. 5, MSP-A; 0/3 experiments) in contrast to exon2 sequences, which were always methylated (Fig. 5, MSP-B; 3/3). We then determined if this methylation pattern leads to a different association of MeCP2 with the genomic DNA at these sequences. ChIP-analyses with anti-MeCP2 antisera revealed that only exon2 sequences, but not exon1 sequences, could be detected after immunoprecipitation (Fig. 5, ChIP-PCR), suggesting that exon2 sequences are higher methylated. While the ChIP data show that methylated CpG-islands of exon2 mediate a better binding of MeCP2 at least in this region of *Nrxn1*, the actual effect on transcriptional activity, inhibiting or enhancing, cannot be inferred directly and depends on the molecular complex forming around MeCP2 at such sites (Chahrour *et al*. 2008).

### Regulatory sequences in *Nlgn* genes

**Figure 6:**
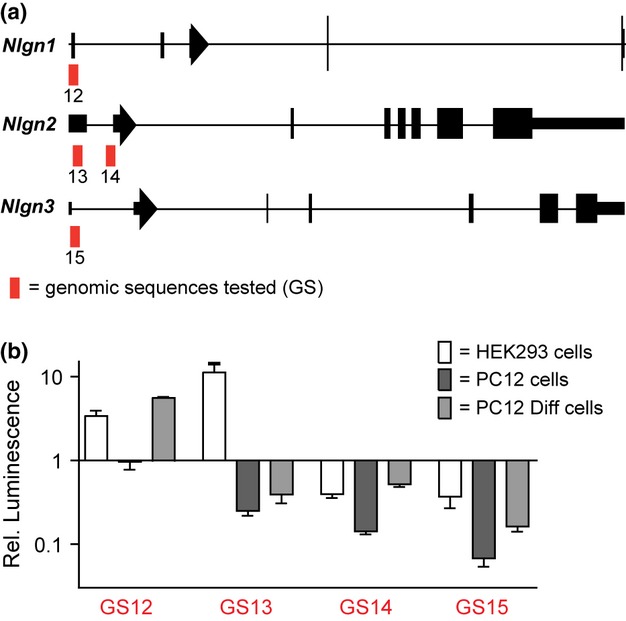
Regulatory sequences in *Nlgn1-3*. (a) Scheme showing exon/intron organization of *Nlgn1-3* genes. Representation is to scale and exons indicated by black bars (arrowheads = translation start, small boxes = UTR). Putative regulatory sequences upstream of transcription start point (TSS) in first exons were predicted by *in silico* analysis (red bars, GS12-15); GS14 marks the TSS of an alternatively spliced *Nlgn2* transcript. (b) Quantitative results from dual luciferase assays in HEK293 (white bars), PC12 (dark gray bars), and nerve growth factor-differentiated PC12 cells (light gray bars) used to probe the regulatory activity of genomic sequences tested (see a). Note that exon1 sequences of *Nlgn2* (GS13) exert differential effects on transcriptional activity in HEK293 compared to PC12 cells. Data are means ± SEM; values for bar graphs are in relation to control vector and blotted logarithmically (> 1 = activation, < 1 = inhibition).

Our observation that *Nlgn* expression was altered in MeCP2 KO brains akin to *Nrxn* (Fig. 1) raised the question if they contain similar regulatory sequences. We focused on regions upstream of the TSS of *Nlgn* and identified by *in silico* analysis putative promoter regions and CpG-islands: GS12 for *Nlgn1*, and GS13 and GS14 for *Nlgn2* (Fig. 6a; Table S3); no CpG-rich sequence was apparent for *Nlgn3* but a corresponding region upstream of the first exons was included in our reporter gene assay. The majority of measurements revealed inhibitory effects on luciferase activity in both HEK293 and PC12 cell lines (Fig. 6b), with the exception of GS12 from *Nlgn1* that showed high activity in HEK293 and differentiated PC12. For *Nlgn2*, we included exon1 and exon2 sequences in our analysis because the first exon is alternatively spliced in some transcripts and both exons contain CpG-islands, a genomic organization highly conserved between different species (Figure S2). We determined that the more upstream sequence (GS13) was highly active in HEK293 but showed strong inhibition in PC12 cells, whereas the sequences preceding exon2 (GS14) exerted only inhibiting activity (Fig. 6b). Since the localization and function of Nlgn2 is specific for inhibitory synapses (Graf *et al*. 2004; Varoqueaux *et al*. 2004; Poulopoulos *et al*. 2009), possibly requiring an intricate regulation of this gene, we analyzed the role of the first two exons of *Nlgn2* in transcriptional activation in detail.

**Figure 7:**
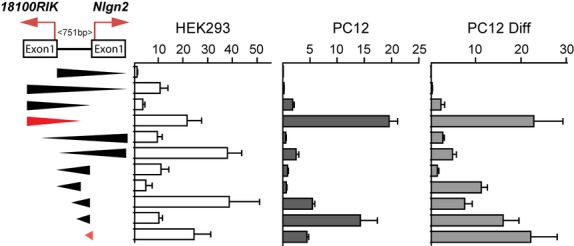
Bidirectional activity driving expression of *Nlgn2* and *RIK18100* genes. The 5′-region of *Nlgn2* is organized in a head-to-head arrangement with an uncharacterized gene (*RIK18100*) on mouse chromosome 11. Their transcription start point (TSS) (red arrows) point in opposite directions and are separated by 751 bp. Arrowheads (left) indicate length, position, and orientation of genomic fragments that were cloned and tested in luciferase assays. Bar graphs show quantitative results for their regulatory activity in HEK293 (white), PC12 (dark gray), and differentiated (light gray) PC12 cells. Strongest transcriptional activity in *Nlgn2* orientation resides upstream of exon1 overlapping with *RIK18100* sequences, whereas optimal activity in *RIK18100* direction is confined to a small fragment upstream of *Nlgn2* exon1 (red arrowheads). Data are means ± SEM (units: relative luminescence); values calculated in relation to control vector (= 1).

We hypothesized that the alternative usage of the different TSS in exon1 and exon2 of *Nlgn2* may have an impact on expression. Sequences including exon2 displayed no or little effect on luciferase activity in HEK293 or in PC12 cells (Table S5). Surprisingly, detailed analysis of the region encompassing exon1 of *Nlgn2* revealed the existence of a second gene on the opposite strand (1810027O10RIK, abbreviated here as *18100RIK*) which resides head-to-head with *Nlgn2* (Fig. 7, scheme; Figure S2). Both genes are separated by only 751 bp between their TSS, representing an arrangement often driven by a bidirectional promoter (Wei *et al*. 2011). We examined expression of *18100RIK* in P7 and P20 mouse brains by RT-qPCR and observed that *18100RIK* mRNA levels mimic those of *Nlgn2*: on P7, expression of both was not significantly changed in MeCP2 KO brains (*Nlgn2*: 0.92 ± 0.12; ns, *18100RIK*: 0.84 ± 0.74; ns), but both genes were up-regulated in KO at P20 (*Nlgn2*: 2.12 ± 0.22; *p *<* *0.0001, *18100RIK*: 5.59 ± 0.61; *p *<* *0.0001), suggesting that the 5′-region of *Nlgn2* mediates a two-sided activity. The predicted amino acid sequence and topology of the putative protein encoded by *18100RIK* are shown in Figure S3 but it is currently unclear, if *18100RIK* transcripts in brain are actually translated or, possibly, serve regulatory purposes.

To determine the sequences driving expression of the two genes, we tested the activity of several overlapping regions in different orientations (Fig. 7; Table S6). In contrast to exon2 fragments of *Nlgn2* (Table S5), we identified several sequences that could significantly increase transcriptional activity in all cell lines, with more pronounced increases in differentiated PC12 cells. We dissected separate genomic fragments for both directions that displayed very strong activating characteristics (Fig. 7, red arrowheads). Since the 3′-ends of these activating sequences are separated by 218 bp, they could be used as independent TSS for *Nlgn2* and *18100RIK*, even in the same neurons. In addition, the higher activity measured in differentiated PC12 compared to native PC12 cells is consistent with the ability of PC12 cells to induce specific gene expression when they differentiate into a more neuron-like phenotype (Mullenbrock *et al*. 2009).

### Methylation of *Nlgn2* and MeCP2 association

**Figure 8:**
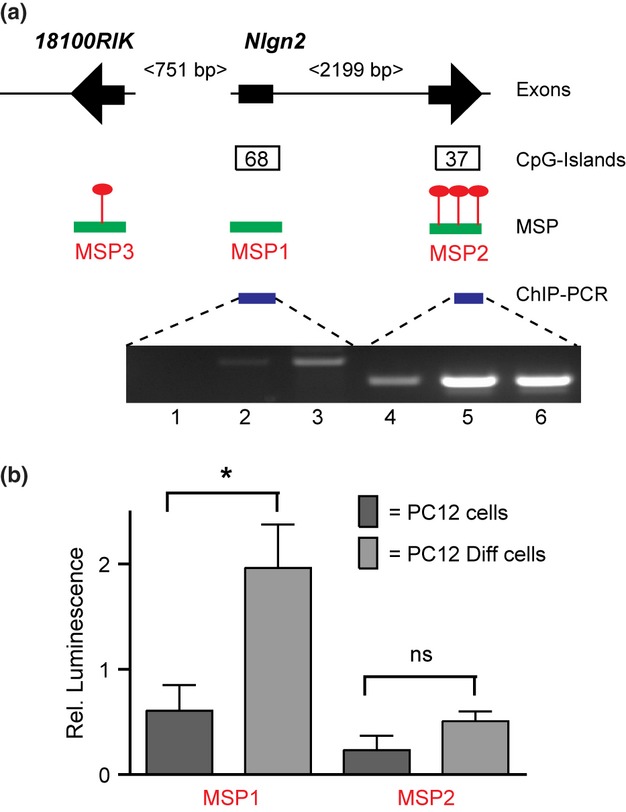
Methylation frequency and methyl-CpG-binding protein2 (MeCP2) binding of *Nlgn2* promoter sequences affect transcriptional activity. (a) Schematic representation of the *Nlgn2*/*18100RIK* head-to-head arrangement (black arrowheads = translation start, black boxes = UTR). Bioinformatics predict that exon1 and exon2 of Nlgn2 contain different numbers of CpG-dinucleotides (numbered boxes). Green lines represent PCR products used for methylation-specific PCRs (MSP1-3) on bi-sulfite converted genomic DNA from PC12 cells (red shapes indicate relative methylation frequency observed). Blue lines denote position of PCR products amplified after chromatin-immunoprecipitation with anti-MeCP2 (ChIP-PCR), shown on sample gel images below (lanes 1, 4: PC12 cells; lanes 2, 5: differentiated PC12; lanes 3, 6: brain DNA as control). (b) Results of luciferase assays using *in vitro*-methylated constructs that correspond to MSP1 and MSP2 fragments. *In vitro* methylation has an effect on MSP1 but not on exon2-containing MSP2, reflecting its strong native methylation shown in (a). Data are means ± SEM; values calculated in relation to control vector (= 1). Significance was determined by Student's *t*-test, significance levels are indicated as *p <* *0.05, ns = non-significant.

To elucidate why exon1 constructs of *Nlgn2* are mostly activating (Fig. 7), whereas exon2 sequences showed an inhibition of reporter gene expression (Table S5), we studied differences in their methylation patterns, using a similar strategy as for *Nrxn1* (Fig. 5). We performed MSP with sequences representing the first (MSP1) and second exon of *Nlgn2* (MSP2), and included the first exon of *18100RIK* (MSP3) as an additional control (Fig. 8a, MSP scheme). MSP2 sequences were strongly methylated in PC12 cells and mouse brain in the majority of experiments (> 70%, nine independent experiments), whereas MSP1 was largely unmethylated (< 5%, eight experiments). In MSP3 samples, we found both methylated and unmethylated PCR products (54% methylated, six experiments), indicating that the distinct methylation patterns of MSP1 and MSP2 are likely to be specific. To test if MeCP2 is preferentially associated with the more methylated region, we performed ChIP-PCR with anti-MeCP2 antisera (Fig. 8a, ChIP-PCR). For this experiment, we used genomic DNA from normal and differentiated PC12 cells because RT-PCR showed that *Nlgn2* is much more abundant than *Nrxn1* (data not shown). PC12 were also chosen based on the hypothesis that differentiation of PC12 cells with NGF induces an increase in MeCP2 expression (Jung *et al*. 2003; Mullenbrock *et al*. 2009), allowing an additional experimental condition. Consistent with the MSP results, we could unequivocally amplify exon2 sequences after immunoprecipitation with anti-MeCP2, suggesting that MeCP2 actually binds to the methylated region around exon2 of *Nlgn2* in both undifferentiated (Fig. 8a, ChIP gel lane 4) and even stronger in differentiated PC12 (Fig. 8a, ChIP gel lane 5). In contrast, we were unable to identify unmethylated exon1 sequences in ChIP-PCR from normal PC12 cells (Fig. 8a, ChIP gel lane 1). In NGF-differentiated PC12 cells, however, we consistently obtained a weak signal (Fig. 8a, ChIP gel lane 2), suggesting that methylation of CpGs is increased in this region or binding increased because of a higher expression of MeCP2, as reported for other neuronal genes (Chahrour *et al*. 2008).

To finally test the possibility that the methylation status itself influences the transcriptional activity of these *Nlgn2* sequences, we performed luciferase assays with *in vitro*-methylated constructs in normal and differentiated PC12 cells. In normal PC12 cells, the experimentally methylated constructs did not show an altered luciferase activity (Fig. 8b, Table S7). However, in differentiated PC12 cells the methylated exon1 construct (containing the MSP1 region) showed a substantially increased activity, whereas the methylated exon2 construct (containing MSP2) performed at the same level in both differentiated and normal PC12 cells (Fig. 8b). Thus, these results demonstrate that different methylation patterns at *Nlgn2* exon1 and exon2 sequences may explain the differences between the TSS, providing the means for an intricate transcriptional regulation of this gene.

## Discussion

We investigated promoter-like genomic sequences in *Nrxn* and *Nlgn* genes that are able to regulate transcriptional activity. Nrxn and Nlgn are pre- and post-synaptic molecules that affect important properties of synapses throughout the brain (Craig and Kang 2007; Missler *et al*. 2011). *Nrxn* and *Nlgn* represent candidates for transcriptional regulation because they are expressed in various isoforms and undergo extensive alternative splicing, leading to differential distribution and localization of distinct variants in subpopulations of neurons (Püschel and Betz 1995; Ullrich *et al*. 1995; Varoqueaux *et al*. 2004; Hoon *et al*. 2011) or even synapses (Song *et al*. 1999; Graf *et al*. 2004; Poulopoulos *et al*. 2009). Recent work has shown that expression levels, and possibly distribution, of specific alternatively spliced Nrxn variants can be adapted by synaptic activity (Iijima *et al*. 2011) and depolarization of neurons (Rozic-Kotliroff and Zisapel 2007). Such adaptation appears meaningful because Nrxn variants that are alternatively spliced at splice site #4 display different binding properties to their *trans*-synaptic partners Nlgn, LRRTM2, and cerebellin (Boucard *et al*. 2005; de Wit *et al*. 2009; Koehnke *et al*. 2010; Joo *et al*. 2011). In addition, splice variant expression may influence synaptic plasticity (Iijima *et al*. 2011). Similarly, regulated distribution of specific isoforms is also relevant for Nlgn because deletion of *Nlgn2* demonstrated that it specifically affects a subset of inhibitory interneurons with distinct physiological properties in the neocortex (Gibson *et al*. 2009). While significant insight into the mechanisms of alternative splicing of Nrxn was provided by identifying the involved RNA-binding proteins Sam68 (Iijima *et al*. 2011), T-STAR (Ehrmann *et al*. 2013), and the heterogeneous nuclear ribonucleoprotein hnRNP L as the binding partner (Rozic *et al*. 2013), little progress was made in identifying regulatory genomic sequences in *Nrxn* or *Nlgn* genes since their initial description (Rowen *et al*. 2002; Tabuchi and Südhof 2002). Our investigation has now revealed three novel aspects of this process:

First, we observed that *Nrxn1-3* and *Nlgn1-3* expression levels are almost simultaneously altered in brains of mice lacking MeCP2 (Figs 2). MeCP2 is a transcriptional regulator specific to the nervous system that can function as enhancer or repressor depending on the context (Adkins and Georgel 2011). Altered expression does not imply that MeCP2 directly regulates *Nrxn* or *Nlgn*. However, we provide experimental evidence that seem to favor this possibility at least for some instances because binding of MeCP2 to genomic DNA is frequently mediated by methylated CpG-dinucleotides clustered in CpG-islands at transcriptionally active regions (Chen *et al*. 2003; Chahrour *et al*. 2008; Skene *et al*. 2010): (i) We demonstrate that CpG-islands occur in some *Nrxn* and *Nlgn* promoter-like regions and can affect transcriptional activity in luciferase reporter gene assays (Figs 6). This method is commonly used to quantify and compare transcriptional activity (Ikeda *et al*. 2002; Tsuritani *et al*. 2007; Chahrour *et al*. 2008; Suter *et al*. 2011), albeit additional effects because of translation rates cannot be excluded. The reporter gene assay was performed in HEK293 cells and in neuronal-like PC12 cells because Nrxn/Nlgn are neuron-specific and differentiation of PC12 with NGF leads to increased expression of MeCP2 (Impey *et al*. 2004; Chahrour *et al*. 2008; Mullenbrock *et al*. 2009). In addition, the strong transcriptional activation of CpG-rich sequences in the 3′-region of *Nrxn2* (Fig. 3) is consistent with the role of 3′-flanking promoters in alternative splicing (Kornblihtt 2005). (ii) *Nrxn1* and *Nlgn2* promoter analyses demonstrate proof-of-concept that MeCP2 binds to these regions depending on the methylation frequency of CpG-islands (Figs 8) and *in vitro*-methylation of exon1 CpG sequences increases transcriptional activity (Fig. 8). Despite these results, it has to be emphasized that *Nrxn* and *Nlgn*, even if some of the regulation is direct, are only two of hundreds of target genes of MeCP2 (Jordan *et al*. 2007; Chahrour *et al*. 2008; Ben-Shachar *et al*. 2009) and that the mode of action of MeCP2 includes different mechanisms (Adkins and Georgel 2011). Likewise, our data do not indicate that *Nrxn* or *Nlgn* gene regulation is solely responsible for the phenotype of MeCP2 KO mice (Medrihan *et al*. 2008; Chao *et al*. 2010) or even accounts for key pathophysiological mechanism in Rett patients.

Second, we identified a novel TSS in *Nrxn1* that may be alternatively spliced in neuronal cells and could serve in the regulation of activity-dependent expression (Iijima *et al*. 2011; Rozic *et al*. 2013). The genomic sequence in this region is highly conserved (Figure S1) and the sequences preceding the first exon exerted a strong activation in our luciferase assay (Figs 5). Interestingly, the transcriptional activity is abolished when fragments from the first intron or the second exon are included, similar to *Nlgn2*, in which the untranslated first exon also exerts a strong activation in the luciferase assay (Figs 8). This arrangement fits the idea that MeCP2 has more activating influence on expression when bound to upstream, mostly untranslated sequences such as promoters and inhibits gene activity when bound to more downstream sequences (Chahrour *et al*. 2008). Recently, usage of such alternatively spliced TSS was confirmed for distribution of *Fxyd1* in different brain regions, another target regulated by MeCP2 (Jordan *et al*. 2007; Banine *et al*. 2011). Thus, while our analysis of promoter-like sequences in *Nrxn1* and *Nlgn2* argues for an involvement of MeCP2 in both cases, the activity profiles may differ.

mRNA and expressed sequence tags (EST) databases suggest that for *Nrxn1* the TSS in exon1 is used for abundant expression of Nrxn1α variants in brain, whereas transcripts starting with exon2 can be found in liver, a tissue with little or no Nrxn1 protein. This distribution is consistent with our finding that exon1 sequences, including only very few CpG, are activating and exon2 sequences, containing many methylated CpG-islands, are inhibiting translational activity (Figs 5). This could indicate that in *Nrxn1*, the methylation of exon2 CpG-islands mediates the classical mode of transcriptional inhibition by MeCP2 complexed to Sin3/HDAC (Adkins and Georgel 2011). To increase expression, the transcription start would be switched to exon1 sequences that exert an increased activity (Figs 4).

Although mRNA and EST databases suggest that expression of *Nlgn2* in brain starts from exon1 but not from exon2, similar to *Nrxn1*, its actual regulation should be different because unlike *Nrxn1* there are CpG-islands in both of the two-first *Nlgn2* exons (Fig. 8). In support, we show that the two *Nlgn2* CpG-rich regions contain different methylation frequency, and that they mediate opposite effects on transcriptional activity: exon2 sequences show little activation, whereas exon1 sequences are active (Fig. 8). Interestingly, we found that activity of exon1 sequences can be enhanced by additional methylation (Fig. 8), a challenging result because generally promoter methylation has been associated with gene silencing, for example, in immune cells (Brenet *et al*. 2011) but also in many studies on MeCP2 (Adkins and Georgel 2011). However, it was shown that MeCP2 binds to methylated CpG-islands more often when they are associated with promoter regions, and that this binding triggers activation of expression (Yasui *et al*. 2007; Chahrour *et al*. 2008), consistent with our data. Since electrical activity is known to influence the methylation pattern via chromatin remodeling (Martinowich *et al*. 2003), we hypothesize that availability of Nlgn2 for inhibitory synapses could be stimulated by increased neuronal/excitatory activity that leads to addition of methyl groups to exon1 CpGs, followed by association of MeCP2 and increased translational activation (Chahrour *et al*. 2008). Co-activation of other transcription factors such as Creb1 (Chahrour *et al*. 2008) could then generate the molecular machinery needed for newly formed or enlarged inhibitory contacts.

Third, we characterized a bidirectional head-to-head configuration in the promoter region of *Nlgn2* and *18100RIK* on opposite genomic strands which is highly conserved (Figure S2). Molecular dissection of the genomic sequences that activate transcription in these opposite directions revealed two crucial regions (Fig. 7). Since these regions are 218 bp apart, we believe that expression of *Nlgn2* and *18100RIK* can be simultaneously regulated as shown for similar promoter arrangements in mice (Kornblihtt 2005) and humans (Wei *et al*. 2011), and could be used for concomitant activation of both genes. Since the expression pattern and function of 18100RIK is completely unknown, and no homologs exist in the databases, it remains unclear at present if the 18100RIK mRNA is translated (Figure S3) or used as a regulatory RNA.

Work on neuronal promoters has revealed that transcriptional regulation in brain extends beyond the action of MeCP2. For example, it was shown that expression of synaptic vesicle proteins is down-regulated by RE1-silencing transcription factor (REST)-binding to methylated promoter sequences which lead to chromatin rearrangement by histone acetylation (Shahbazian *et al*. 2002b; Ekici *et al*. 2008). Along this line, a binding site for repressor element 1 (RE1/NRSE) has previously been identified in *Nrxn3* (Bruce *et al*. 2004). The activity of REST may also influence the expression of *Nlgn2* because REST leads to an activity-dependent developmental switch from excitatory to inhibitory synapses (Varoqueaux *et al*. 2004; Yeo *et al*. 2009). These results suggest that different regulatory pathways may intersect as expression patterns of *Nrxn* genes are activity dependent (Shapiro-Reznik *et al*. 2012) and expression of REST is adjusted by MeCP2 (Ballas *et al*. 2005). Thus, expression of *Nrxn* and *Nlgn* is likely not affected by MeCP2 alone but is influenced by a larger group of transcriptional regulators which tune the levels of these functionally important molecules during development, plasticity and maintenance of synapses. The quest for the mechanisms involved has just started.

## Abbreviations

ChIP: chromatin-immunoprecipitation
KO: knockout
MeCP2: methyl-CpG-binding protein2
MSP: methylation-specific PCR
NGF: nerve growth factor
TSS: transcription start point

## Supporting information

**Table S1**. RT-qPCR analysis of *Nrxn* and *Nlgn* transcript levels. **Table S2.** Protein levels of Nlgn in MeCP2 KO brains. **Table S3.** Transcriptional activity of regulatory sequences in *Nrxn* and *Nlgn* genes. **Table S4**. Transcriptional activity of sequences in the 5'-region of *Nrxn1*. **Table S5**. Transcriptional activity of exon2 sequences in *Nlgn2*. **Table S6**. Transcriptional activity of sequences from the head-tohead region of *Nlgn2* and *18100RIK*. **Table S7**. Transcriptional activity of methylated sequences from the *Nlgn2* gene. **Figure S1**. Genomic organization and conservation of the transcriptional start site of *Nrxn1*. **Figure S2**. Conservation of the head-to-head organization of the *Nlgn2/18100RIK* locus. **Figure S3**. Predicted protein sequence and domain organization of 1810027O10RIK.
