# Supplementary material for: Promoter-like sequences regulating transcriptional activity in neurexin and neuroligin genes
Source: J Neurochem. 2013 Aug 21;127(1):36–47. doi: 10.1111/jnc.12372 (PMC3910144; doi:10.1111/jnc.12372)
Supplement: Supplementary file 1 — Table S1. RT-qPCR analysis of Nrxn and Nlgn transcript levels. Table S2. Protein levels of Nlgn in MeCP2 KO brains. Table S3. Transcriptional activity of regulatory sequences in Nrxn and Nlgn genes. Table S4. Transcriptional activity of sequences in the 5'-region of Nrxn1. Table S5. Transcriptional activity of exon2 sequences in Nlgn2. Table S6. Transcriptional activity of sequences from the head-tohead region of Nlgn2 and 18100RIK. Table S7. Transcriptional activity of methylated sequences from the Nlgn2 gene. Figure S1. Genomic organization and conservation of the transcriptional start site of Nrxn1. Figure S2. Conservation of the head-to-head organization of the Nlgn2/18100RIK locus. Figure S3. Predicted protein sequence and domain organization of 1810027O10RIK. [file jnc0127-0036-sd1.pdf]

Supporting Information for:

Promoter-like sequences regulating transcriptional activity in neurexin and neuroligin genes

Fabian Runkel, Astrid Rohlmann, Carsten Reissner, Stefan-Martin Brand, and Markus Missler*

* Corresponding Author (e-mail: Markus.Missler@uni-muenster.de)

This file includes:

- Supplementary Tables S1 to S7
- Supplementary Figures S1 to S3 (including legends)

Supplementary Table S1. RT-qPCR analysis of *Nrxn* and *Nlgn* transcript levels

Isoform	Forward Primer	Reverse Primer	Exon	Ratio (KO/WT) at P7	Ratio (KO/WT) at P20
Nrxn1α	CCCCAGCACAGCCGACCTTC	GCAAGTCGCGATAATTCCAGCCT	8-9	0.49±0.06, P<0.0001	1.99±0.17, P<0.0001
Nrxn2α	CTACCTTCTGCTGGACATGGGCTCC	GCGTGCTGCGGCTGTTCACA	8-9	0.62±0.05, P<0.0001	2.14±0.16, P<0.0001
Nrxn3α	GCACCATCAAAGTGAAGGCCACTC	GCCCAGATACATGTCCCCCTCCA	8-9	0.86±0.08, P=0.0025	1.83±0.48, P=0.0131
Nrxn1β	CCATGGCAGCAGCAAGCATCATTCA	CGTGTACTGGGGCGGTCATTGGGA	1-2	0.55±0.06, P<0.0001	1.48±0.10, P=0.001
Nrxn2β	GTCTCGTCCAGCCTCAGCACCACC	CGTGTACTGGGCCGGTCATTGGGA	1-2	0.66±0.03, P<0.0001	1.66±0.02, P=0.0003
Nrxn3β	CTCCGGGATCTCACTCTCAGCAGG	GATGAGGCCACCGCTTTTCCCAA	1-2	0.54±0.11, P<0.0001	2.13±0.21, P=0.0006
Nlgn1	CACAGTCAACTATCGGCTTGGGGTAC	CAAACACAGTGATTCGCAAGGGGTC	5-6	0.54±0.21, P=0.0037	1.96±0.33; P=0.0020
Nlgn2	CCATTTCCAGCTGGTCTGTCAACTAC	CCAAAGGCAATGTGGTAGCGGG	5-6	0.92±0.12, ns	2.12±0.22, P=0.0001
Nlgn3	CAGGCAACATGATTGATGGCAGTGT	GAAGGCAATATTCTCACTCACCCAGCGA	4-5	0.66±0.08, P=0.0163	1.94±0.08, P=0.0018

Exon-spanning RT-qPCRs were performed between the indicated exons. Note that oligonucleotides for *Nlgn1* and *Nlgn2* only exist in the mRNA sequence. Values describe the relative expression of transcripts in wild-type (WT) vs. MeCP2-knockout (KO) samples from day P7 and P20. As described in Material and methods, values below 1 indicate that expression in the knockout was decreased, values above 1 indicate an increased expression in the knockout sample. Significance levels were tested with Student´s t-test, exact *P* values indicated, ns = not significant

Supplementary Table S2. Protein levels of Nlgn in MeCP2 KO brains

Protein (Age)	Relative expression[a]	n[b]	P value[b]
Nlgn1 (P7)	1.57±0.14	10	P=0.0024
Nlgn1 (P20)	0.66±0.12	9	P=0.0183
Nlgn2 (P7)	1.33±0.16	3	ns
Nlgn2 (P20)	0.57±0.05	10	P<0.0001
Nlgn3 (P7)	1.02±0.15	5	ns
Nlgn3 (P20)	0.67±0.08	4	P=0.0288

Quantitative immunoblots of brain lysates were used to determine the protein levels of Nlgn1-3 in WT and MeCP2 KO mice at age P7 and P20.

[a] Expression of protein is calculated as relative luminescence signal in KO/signal in WT.

[b] Number (n) of blots quantified for each protein; significance was tested with Student´s t-test; ns = not significant.

Supplementary Table S3. Transcriptional activity of regulatory sequences in *Nrxn* and *Nlgn* genes

Region	Primer pairs (5´-3´)	Genomic localization	HEK293 (%)	PC12 (%)	PC12 Diff (%)
Nrxn1					
GS1	CGCGGCTAGCGCGTAATTTAAAGCCACAGATTCC	chr17:91,491,931-91,493,228	358±75, P=0.0745	600±73, P=0.0205	672±137, P=0.1504
	CGCGAGATCTGCCTGGTTATTCCCCTTAGC				
GS2	CGCGGCTAGCATTTGGGATTGGGACAGAACC	chr17:91,486,713-91,489,051	142±26, P=0.2423	5±1, P<0.0001	14±8, P<0.002
	CGCGAAGCTTAACCACTGTTTATTTACCTGCTTTCC				
GS3	CGCGGCTAGCGAGGGTAGGTAGCTCACTGTACTGC	chr17:90,853,524-90,855,873	43±4, P=0.0056	4±1, P<0.001	4±0.3, P=0.002
	CGCGAAGCTTTCTCTTCTCACCCTCTTTTGTTCC				
Nrxn2					
GS4	CGCGGCTAGCGTATGCACAAGTATGCAGGTTATGG	chr19:6,417,406-6,419,731	96±12, P=0.7547	12±2, P=0.0006	28±3, P=0.0304
	CGCGCTCGAGTCTACCCTTCCAGTACTCTGTGACC				
GS5	CGCGCTCGAGCAAGGGTGACCAGGAGTAATGG	chr19:6,426,328-6,428,974	37±8, P=0.0046	9±1, P=0.0002	11±0.1, P=0.0001
	CGCGAAGCTTCAACGGGTTTACTCTGGAGTCG				
GS6	CGCGGCTAGCGCTTCCTTGTAACTGTAGCTCTTGG	chr19:6,480,064-6,482,467	35±3, P=0.0046	22±2, P=0.0006	59±10, P=0.0585
	CGCGAAGCTTGCGCTCAGAGTACACACATAAACC				
GS7	CGCGCTCGAGGAGAGAGAGAGTGTGTCTCCAGTGC	chr19:6,496,832-6,499,225	283±30, P=0.0264	99±12, P=0.9493	93±27, P=0.8500
	CGCGAAGCTTCTTGGAGACAGAGGGTATGAAGG				
GS8	CGCGGCTAGCCCAGAATCCTAAGGGACAAACC	chr19:6,530,283-6,532,616	696±68, P=0.0031	414±65, P=0.0071	710±59, P=0.0617
	CGCGAAGCTTGAGGGTAGGGACAAGAGACAGC				
Nrxn3					
GS9	CGCGGGTACCAAACCAACTTGCATGAGTTAAATGG	chr12:89,958,571-89,961,016	4±0.3, P<0.0001	8±3, P=0.0011	18±4, P=0.0298

	CGCGCTCGAGTCCTGCACTCCTGTACAATAAAAGG				
GS10	CGCGGCTAGCTCTTAGGTTATCTGCTGATTTTCTGC	chr12:90,497,668-90,499,996	13±4, P=0.0022	5±2, P=0.0004	12±1, P=0.0038
	CGCGAAGCTTAATGCTTAAGCTGTGCTTAGAGAGG				
GS11	CGCGCTCGAGGTGTGTGTGTATAGTGTGCCTACCC	chr12:91,049,717-91,052,165	113±20, P=0.5908	5±1, P=0.0003	10±4, P=0.0296
	CGCGAGATCTCTGGAATCTACCTGAAAGACAGTGG				
Nlgn1					
GS12	CGCGGCTAGCCAGAAAGTGACATCTTGACTTGAACC	chr3:26,230,046-26,232,469	325±52, P=0.005	98±19, P=0.9118	564±15, P=0.0213
	CGCGCTCGAGTACAAGGCTGTCAGGAGTAGTGAGC				
Nlgn2					
GS13	CGCGGGTACCGACCTACACTTGACTGCCTTCC	chr11:69,649,612-69,652,566	1079±317, P=0.023	25±3, P=0.0017	39±8, P=0.0109
	CGCGGCTAGCTTTATTCCAGAGATGAGGACACAGG				
GS14	CGCGGCTAGCAGTGGCGAGATTATATGTCAAAAGG	chr11:69,833,159-69,835,460	40±4, P=0.0001	57±9, P=0.0441	52±4, P=0.0528
	CGCGCTCGAGCGTTGCCGAGGTAACATTGC				
Nlgn3					
GS15	CGCGGCTAGCGGAGCAGAGCTTGCCTGACG	chrX:98,494,304-98,496,672	37±10, P=0.0035	7±1, P=0.0002	16±2, P=0.0007
	CGCGAAGCTTCTCTCCTTGACATTTCTCCTCAGC				

Genomic fragments (GS1-15; see Figures 3 and 6) including putative regulatory sequences of *Nrxn* and *Nlgn* isoforms were cloned in a luciferase vector, using the indicated primer pairs. The genomic localization of the sequences tested is indicated. Results of the reporter gene assay in HEK293 cells, PC12 and differentiated PC12 cells (PC12 Diff) represent percent of vector control (= 100%). Each region was measured in 3-6 independent experiments. Significance levels were tested with Student´s t-test; and *P* values listed.

Supplementary Table S4. Transcriptional activity of sequences in the 5´-region of *Nrxn1*

No.	Primer pairs (5´-3´)	Genomic locus	HEK293 (%)	PC12 (%)	PC12 Diff (%)
1	CGCGGCTAGCGCGTAATTTAAAGCCACAGATTCC				
	CGCGAGATCTGCCTGGTTATTCCCCTTAGC	chr17:91,491,941-91,493,218	358±75, P=0.0754	600±73, P=0.0205	672±138, P=0.1504
2	CGCGGCTAGCGCCACAGATTCCAGACTTCTATCC				
	CGCGAGATCTCAGTTGGTTGTATGTTGGTGATGC	chr17:91,488,883-91,493,206	102±10, P=0.8483	158±17, P=0.0266	252±50, P=0.2010
3	CGCGCGGTACCCCCTTCTCTTTCAGAAGGACAGC				
	CGCGAAGCTTCGACAGGCAGAGGAGACAGC	chr17:91,488,016-91,492,218	18±0.5, P<0.0001	35±0.3, P=0.0032	29±0.7, P=0.0065
4	CGCGGCTAGCGCAGGTAAAGGAGCTCAATTTGG				
	CGCGAGATCTCAGTTGGTTGTATGTTGGTGATGC	chr17:91,488,883-91,491,942	130±7, P=0.0088	47±7, P=0.0059	46±8, P=0.1007
5	CGCGGCTAGCAGGAGGGCACAAGAGAGTAGGC				
	CGCGAGATCTATTCAACCATTCCAAGCAAACACG	chr17:91,489,950-91,490,907	102±30, P=0.3430	76±22, P=0.3430	151±22, P=0.1499
6	CGCGGCTAGCAGGAGGGCACAAGAGAGTAGGC				
	CGCGAGATCTCAGTTGGTTGTATGTTGGTGATGC	chr17:91,488,883-91,490,907	38±2, P=0.0009	26±3, P=0.0014	58±9, P=0.1306
7	CGCGGCTAGCGTAGGGTGACGTGTTTGCTTGG				
	CGCGAGATCTCAGTTGGTTGTATGTTGGTGATGC	chr17:91,488,883-91,489,982	156±17, P=0.0847	68±12, P=0.1122	53±10, P=0.1309
8	CGCGGCTAGCATTTGGGATTGGGACAGAACC				
	CGCGAAGCTTAACCACTGTTTATTTACCTGCTTTCC	chr17:91,486,723-91,489,041	127±24, P=0.3526	5±0.8, P<0.0001	14±4, P=0.0002
9	CGCGCGGGTACCAACTGACATGCTGGAGTCTTAGGG				
	CGCGAAGCTTCGACAGGCAGAGGAGACAGC	chr17:91,488,016-91,488,887	30±4, P=0.0032	37±4, P=0.0455	11±3, P=0.0245
10	CGCGCGGGTACCAACTGACATGCTGGAGTCTTAGGG				
	CGCGCGGCTAGCCCTCGAGCAGTCGCATACGG	chr17:91,487,326-91,488,887	51±13, P=0.0144	61±26, P=0.2347	54±19, P=0.2530

Several overlapping genomic fragments around exon1 and exon2 of the *Nrxn1* gene were cloned in a luciferase vector, using the indicated primer pairs (No. refer to the order of fragment shown in Figure 4). The genomic localization of the sequences tested is indicated. Results of the luciferase assays in HEK293 cells, PC12 and differentiated PC12 cells (PC12 Diff) represent percent of vector control (= 100%). Each region was measured in 3-6 independent experiments. Significance levels were tested with Student´s t-test; and *P* values listed.

Supplementary Table S5. Transcriptional activity of exon2 sequences in *Nlgn2*

No.	Oligonucleotides (5´-3`)	Genomic locus	HEK (%)	PC12 (%)
1	CGCGGCTAGCGAAAAGCCCAGCGTCAGC<br>CGCGCTCGAGCCCCTGACATCCCCTTCC	chr11:69,648,367-69,649,588	44±5, P=0.0013	42±5, P=0.0077
2	CGCGGCTAGCAGTGGCGAGATTATATGTCAAAAGG<br>CGCGCTCGAGCGTTGCCGAGGTAACATTGC	chr11:69,646,651-69,648,972	40±4, P=0.0001	57±9, P=0.0441
3	CGCGGCTAGCCGTTGCCGAGGTAACATTGC<br>CGCGCTCGAGCCCCTGACATCCCCTTCC	chr11:69,648,367-69,648,972	22±3, P<0.0001	31±8, P=0.0036
4	CGCGGCTAGCATGGGGACAGAAGGTACTGG<br>CGCGCTCGAGCCCCTGACATCCCCTTCC	chr11:69,648,367-69,648,766	149±26, P=0.1488	120±13, P=0.2333

Several overlapping genomic fragments in the vicinity of exon2 of the *Nlgn2* gene were cloned in a luciferase vector, using the indicated primer pairs. The genomic localization of the sequences tested is shown. Results of the luciferase assays in HEK293 cells and PC12 cells represent percent of the vector control (= 100%). Each region was measured in 3-6 independent experiments. Significance levels were tested with Student´s t-test; and *P* values listed.

Supplementary Table S6. Transcriptional activity of sequences from the head-to-head region of *Nlgn2* and *18100RIK*

**No.**	**Primer pairs** (5´-3´)	**Genomic locus**	**HEK293 (%)**	**PC12 (%)**	**PC12 Diff (%)**
1	CGCGGCTAGCTTTATTCCAGAGATGAGGACACAGG				
	CGCGGGTACCATCAGGGCTATGTTCATTTCAGAGC	chr11:69,649,612-69,651,979	147±24, P=0.1877	10±2, P=0.0006	14±3, P=0.0732
2	CGCGGGTACCGACCTACACTTGACTGCCTTCC				
	CGCGGCTAGCTTTATTCCAGAGATGAGGACACAGG	chr11:69,649,612-69,652,566	1079±317, P=0.023	25±3, P=0.0017	39±8, P=0.0109
3	CGCGGGTACCGACCTACACTTGACTGCCTTCC				
	CGCGGCTAGCAGTGAAAGGGGTGGGAGTGG	chr11:69,651,382-69,652,566	382±78ns, P=0.0683	182±27, P=0.0944	283±90, P=0.1543
4	CGCGGGTACCGACCTACACTTGACTGCCTTCC				
	CGCGGCTAGCCGCTCCTAATTGGTTCTAGTTTCTCC	chr11:69,651,847-69,652,566	2172±586, P=0.0144	1959±151, P=0.025	2286±642, P=0.0036
5	CGCGGGTACCTTTATTCCAGAGATGAGGACACAGG				
	CGCGGCTAGCGACCTACACTTGACTGCCTTCC	chr11:69,649,612-69,652,566	971±198, P=0.0481	57±9, P=0.0418	276±32, P=0.1133
6	CGCGGCTAGCTTTATTCCAGAGATGAGGACACAGG				
	CGCGAAGCTTATCAGGGCTATGTTCATTTCAGAGC	chr11:69,649,612-69,651,979	3798±576, P=0.0077	284±40, P=0.0448	488±85, P=0.0444
7	CGCGGGTACCAGTGAAAGGGGTGGGAGTGG				
	CGCGGCTAGCGACCTACACTTGACTGCCTTCC	chr11:69,651,382-69,652,566	1114±322, P=0.0294	89±19, P=0.6278	152±38, P=0.4004
8	CGCGGGTACCCGCTCCTAATTGGTTCTAGTTTCTCC				
	CGCGGCTAGCGACCTACACTTGACTGCCTTCC	chr11:69,651,847-69,652,566	502±259, P=0.2616	68±9, P=0.0684	1128±127, P=0.004
9	CGCGGGTACCAGTGAAAGGGGTGGGAGTGG				
	CGCGAAGCTTATCAGGGCTATGTTCATTTCAGAGC	chr11:69,651,382-69,651,979	3872±1225, P=0.0349	548±45, P=0.0099	759±163, P=0.0273
10	CGCGGGTACCAGTGAAAGGGGTGGGAGTGG				
	CGCGGCTAGCTTGAGGGTGGGACTAGAGGA	chr11:69,651,382-69,651,840	1030±148, P=0.0245	1429±310, P=0.0303	1605±350, P=0.00232
11	CGCGGGTACCAGTGAAAGGGGTGGGAGTGG				
	CGCGGCTAGCCCACGGATTTCTAAGCTCCA	chr11:69,651,382-69,651,650	2447±676, P=0.0217	449±31, P=0.0038	2680±494, P=0.0088

Several overlapping genomic fragments between the first exons of the *Nlgn2* and *18100RIK* genes were cloned in a luciferase vector, using the indicated primer pairs (No. refer to the order of fragment shown in Fig. 7). The genomic localization of the sequences tested is shown. Results of the luciferase assays in HEK293 cells, PC12 and differentiated PC12 cells (PC12 Diff) represent percent of the vector control (= 100%). Each region was measured in 3-6 independent experiments. Significance levels were tested with Student´s t-test; and *P* values listed.

Supplementary Table S7. Transcriptional activity of methylated sequences from the *Nlgn2* gene

No.	Oligonucleotides (5´-3`)	Genomic locus	PC12 (%)	PC12 Diff (%)
MSP1	CGCGGGTACCGACCTACACTTGACTGCCTTCC<br>CGCGGCTAGCTTTATTCCAGAGATGAGGACACAGG	chr11:69,649,612-69,652,566	61±42	197± 72<br>P=0.0485
MSP2	CGCGGCTAGCAGTGGCGAGATTATATGTCAAAAGG<br>CGCGCTCGAGCGTTGCCGAGGTAACATTGC	chr11:69,833,159-69,835,460	23±24	51±16,<br>P=0.1807

Genomic fragments from the 5´-region of *Nlgn2* were cloned in a luciferase vector, using the indicated primer pairs (No. refer to the fragments shown in Fig. 8). The sequences used are the same as in GS13 (MSP1) and GS14 (MSP2) displayed in Fig. 6. The DNA was methylated *in vitro* as described in Material and methods before transfection into PC12 and differentiated PC12 (PC12 Diff) cells. The genomic localization of the sequences tested is indicated. Results of the luciferase assays represent percent of the methylated vector control. Each region was measured in 3 independent experiments. Statistic and significance levels compared the results from both cells lines, and were tested with Student´s t-test; and *P* values listed.

Supporting Information

Supplementary Figures S1 to S3

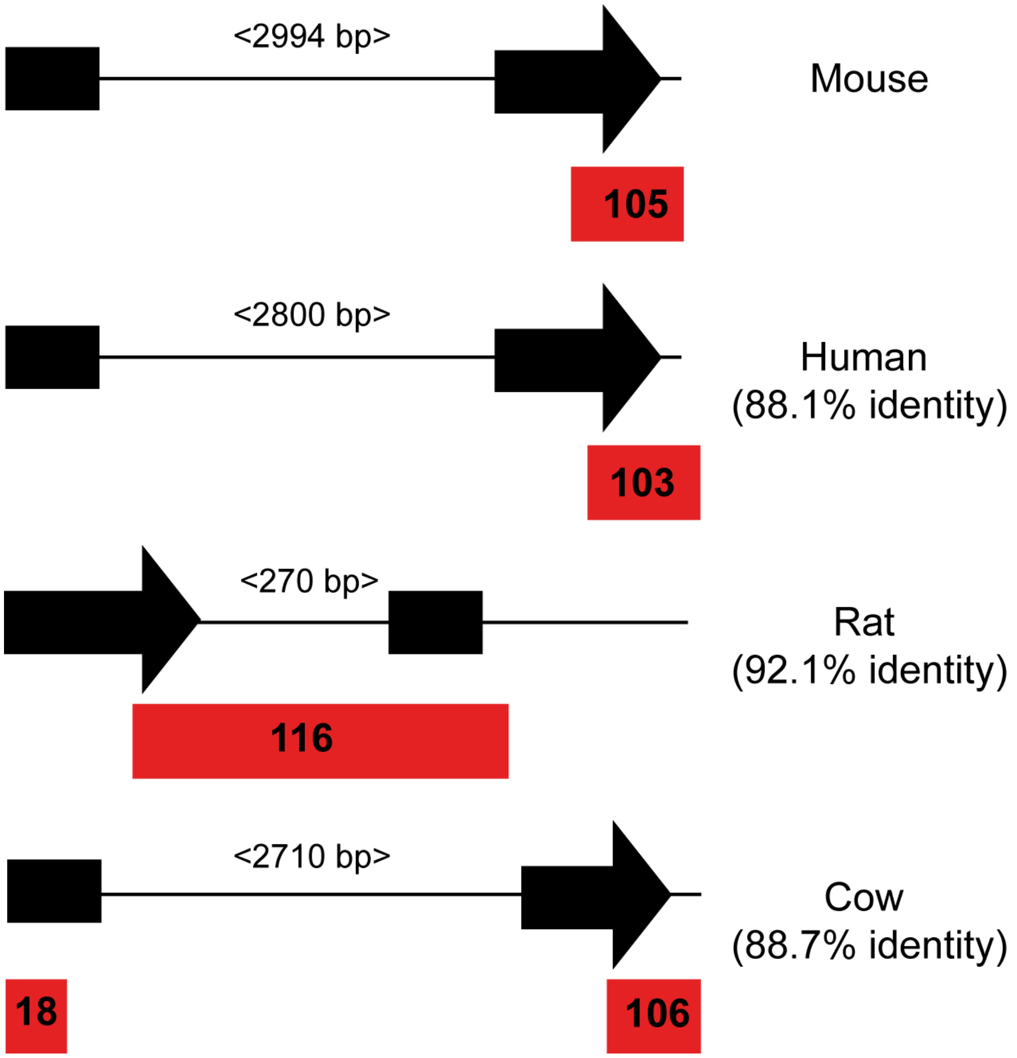

Figure S1. Genomic organization and conservation of the transcriptional start site of *Nrxn1*.

The genomic organization of the first two exons of *Nrxn1* is schematically shown for mouse (NCBI37/mm9), human (GRCH37/hg19), rat (RGSC 5.0/rn5) and cow (Baylor Btau_4.6.1/bosTau7). Distances between exons are given in base pairs and number of CG-dinucleotides are indicated in red squares. Black boxes represent exons and arrowheads the translation start points. Identity values were calculated for the displayed region compared to the mouse genome assembly NCBI37/mm9 from July 2007.

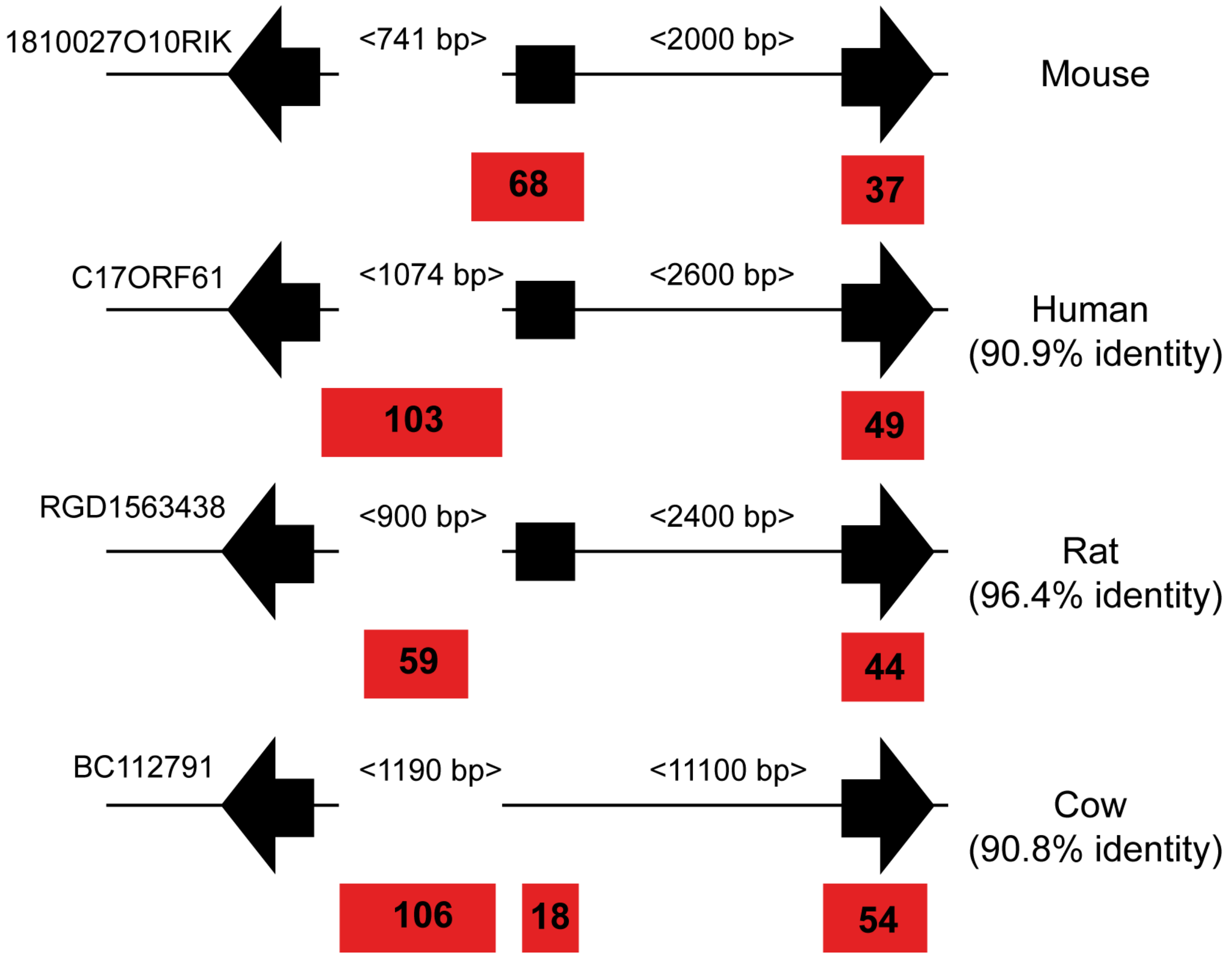

Figure S2. Conservation of the head-to-head organization of the *Nlgn2/18100RIK* locus.

The genomic organization of the Nlgn2/18100RIK locus is schematically shown for mouse (NCBI37/mm9), human (GRCH37/hg19), rat (RGSC 5.0/rn5) and cow (Baylor Btau_4.6.1/bosTau7). Distances between exons are given in base pairs and number of CG-dinucleotides are indicated in red squares. Black boxes represent exons and arrowheads the translation start points. Identity values were calculated for the displayed region compared to the mouse genome assembly NCBI37/mm9 from July 2007.

A)

MAGVGAAFRRLGALSGAGALGLASYGAHGAQFPDAYG
KELFDKANKHHFLHSLALLGVPSCRKPVWAGLLLASGTT
LFCTSFYYQALSGDTSIQTLGPVGGSLLILGWLALAF

B)

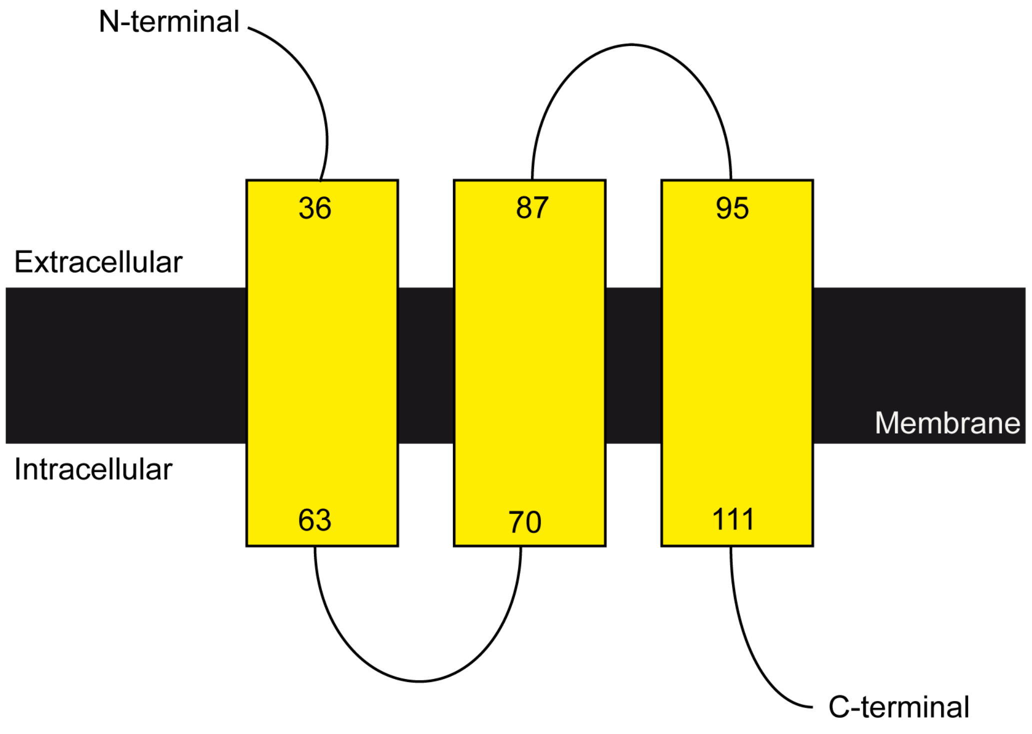

Figure S3. Predicted protein sequence and domain organization of 1810027O10RIK.

(A) 1810027O10RIK mRNA has a complete length of 435 bp and contains an open reading frame, predicted to code for 113 amino acids (shown above in one-letter code). *In silico* analysis of the aa sequence revealed a hypothetical signal peptide (red) and three transmembrane domains (green). (B) Deduced model of the protein spanning the plasma membrane with very short internal and external loops (black).
